# Multisystem Inflammatory Syndrome in Children (MIS-C), a Post-viral Myocarditis and Systemic Vasculitis—A Critical Review of Its Pathogenesis and Treatment

**DOI:** 10.3389/fped.2020.626182

**Published:** 2020-12-16

**Authors:** Jeremy C. McMurray, Joseph W. May, Madeleine W. Cunningham, Olcay Y. Jones

**Affiliations:** ^1^Department of Pediatrics, Walter Reed National Military Medical Center (WRNMMC), Bethesda, MD, United States; ^2^Division of Pediatric Cardiology, Walter Reed National Military Medical Center (WRNMMC), Bethesda, MD, United States; ^3^Department of Pediatrics, Uniformed Services University of the Health Sciences, Bethesda, MD, United States; ^4^Department of Microbiology and Immunology, University of Oklahoma Health Sciences Center, Oklahoma City, OK, United States; ^5^Division of Pediatric Rheumatology, WRNMMC, Bethesda, MD, United States

**Keywords:** MIS-C, myocarditis, vasculitis, coronary artery aneurysm, IL-1 (interleukin-1)

## Abstract

MIS-C is a newly defined post-viral myocarditis and inflammatory vasculopathy of children following COVID-19 infection. This review summarizes the literature on diagnosis, parameters of disease severity, and current treatment regimens. The clinical perspective was analyzed in light of potential immunopathogenesis and compared to other post-infectious and inflammatory illnesses of children affecting the heart. In this paradigm, the evidence supports the importance of endothelial injury and activation of the IL-1 pathway as a common determinant among MIS-C, Kawasaki disease, and Acute Rheumatic fever.

## Introduction

In the midst of a global pandemic of COVID-19 with the origin of its first cases identified in December 2019 in Wuhan City in China (1), most children in the early days of the pandemic were either asymptomatic or mildly symptomatic with fever and cough; this changed with the emergence of multisystem inflammatory syndrome in children (MIS-C) (2–4). Since late April 2020, children began presenting with fever, gastrointestinal symptoms, and features of myocarditis, including some with coronary artery aneurysms (CAAs), as clustered cases in the Western hemisphere (5). Initially, there was uncertainty whether these children were manifestations of Kawasaki disease (KD) or toxic shock syndrome (TSS), or instead represented a phenomenon temporally-related to the ongoing COVID-19 pandemic. Soon it was recognized that this was a new pediatric inflammatory syndrome caused by Severe Acute Respiratory Syndrome Coronavirus 2 (SARS-CoV-2). Interestingly, cases of MIS-C were emerging around 4–5 weeks on average following the peak incidence of COVID-19 in each particular region (6–10). Furthermore, as shown in Table 1, most children had negative COVID-19 testing by nasopharyngeal reverse-transcriptase polymerase chain reaction (RT-PCR), but positive serum levels of anti-SARS-CoV2 antibodies (6, 7, 11–23). Collectively, these results suggested that MIS-C is a post-viral inflammatory disease, rather than an unremitting infection. The current definition of MIS-C was established by the Centers for Disease Control and Prevention in May 2020 and involves the following: an individual <21 years presenting with a fever for > 24 h, laboratory evidence of inflammation, and evidence of severe illness requiring hospitalization, with multisystem involvement of >2 organs (cardiac, renal, respiratory, hematologic, gastrointestinal, dermatologic or neurological); and no alternative diagnoses; and positive recent or current SARS-CoV-2 infection or exposure to a suspected or confirmed COVID-19 case (24).

**Table 1:** MIS-C clinical presentation data from a systematic review of 15 case series/reports (6, 7, 11–23).

**Confirmed MIS-C patients (%)**
Number of patients (*n*)	255
Median age (years)	5–11.5
SARS-CoV-2 RT-PCR Positive	32.2%
SARS-CoV-2 IgG and/or IgM Positive	95.1%
Gastrointestinal symptoms	91.9%
Shock	67.8%
Myocarditis	75.3%
Coronary artery aneurysms	17.5%

The following is a summary of the current knowledge on MIS-C in conjunction with evidence-based medicine on selected conditions. The objective of this review is to offer unique perspectives toward patient assessment and management using known research including clinical and laboratory data on MIS-C and MIS-C like conditions.

## SARS-CoV-2, A New Viral Strain of Betacoronaviruses With Tropism to Myocardial and Endothelial Cells

### SARS-CoV-2 (i.e., COVID-19) Is the Newest Outbreak of Betacoronaviruses After SARS-CoV and MERS-CoV

Betacoronaviruses are one of the four genera (alpha, beta, gamma, and delta) of coronaviruses composed of enveloped capsid with spike protein carrying positive-sense, single-stranded RNA. They generally cause a mild respiratory tract infection, often known as the “common cold.” However, in some cases, they can lead to life threatening illnesses among infants, the immunocompromised, and the elderly. SARS-CoV-2 is a newly discovered betacoronavirus sharing about 79.6% homology with severe acute respiratory syndrome coronary virus (SARS-CoV) and 50% homology with Middle East respiratory syndrome coronavirus (MERS-CoV), which are other strains in the same genera (25). Similar to SARS-CoV-2, SARS-CoV and MERS-CoV were involved in major outbreaks of severe respiratory illnesses (26, 27) infecting about 8,000 people in 2003 and 2,500 in 2012 people, respectively. In both instances, pediatric cases constituted <2% infected with an overall mild course; among 135 reported pediatric cases of SARS-CoV, there were no fatalities (28). Out of 14 pediatric MERS-CoV cases, at a mean age of 99 months, 2 patients died after developing severe respiratory symptoms and multi-organ dysfunction (29).

SARS-CoV-2 has demonstrated higher rates of transmission, which have led to the rapid spread and the current pandemic. As of October 18, 2020, there were 40,118,333 cases and 1,114,749 deaths worldwide per published data from the World Health Organization (WHO) (30). Overall, children below the age of 18 years constitute only around 2% of all COVID-19 cases, based on reports from the US (2%), China (2.2%), Italy (1.2%), and Spain (0.8%) (31). A large cohort study in China of over 2000 pediatric patients who were positive with COVID-19 revealed a wide variation in the clinical presentation, where 4% were asymptomatic; 51% with mild symptoms such as fever, cough, myalgia, or fatigue; 5% with signs of severe disease including dyspnea, and hypoxia; and 0.6% presenting with the critical disease of respiratory failure, acute respiratory distress syndrome, shock, and/or multi-organ dysfunction (32). An inverse relationship between age and incidence of severity of disease was observed with 10.6% of young infants <1 year of age as opposed to 3% of children 16 and older developing severe and critical symptoms (32).

### Viral Tropism of Myocardial and Endothelial Cells

All three betacoronaviruses, show tropism to many cell types in the body including epithelial cells in the linings of the lung and gastrointestinal tract as well as cardiovascular involvement in myocardial and endothelial cells. The viral entry to the cell is facilitated by binding of a spike protein to the angiotensin-converting enzyme 2 (ACE2) receptor for SARS-CoV and SARS-CoV-2, and CD26 for MERS (33, 34). The incubation period for SARS-CoV-2 is very similar to SARS-CoV and MERS-CoV with a mean of about 6.4 days until onset of symptoms (35). Unlike SARS-CoV and MERS-CoV, which have more viral copies present in the lower respiratory tract, SARS-CoV-2 has a predominant viral load in the upper airway (36). Additionally, viral loads of SARS-CoV-2 are highest at symptom onset and decline after 5–6 days as opposed to SARS-CoV, which has a peak of the viral load around 6–11 days after symptom onset (36). The correlation of this observation to the differences in viral genomes or host immune responses is not known.

### Is There a Correlation Between Viral Load and the Severity of COVID-19?

It is important to point out that, to date, there has not been a clear correlation of viral load with the symptomology or the severity of COVID-19. A recent report on the viral load measurement by PCR of initial nasopharyngeal swab samples among patients admitted to the hospital showed that those who were critically ill had a higher viral load compared to those who had an uncomplicated admission (37). Overall, up to 45% of children with COVID-19 are asymptomatic (38). Yet, young children carry significantly higher SARS-CoV-2 viral load than adults (39, 40). It is reasonable to speculate that children experience viral tropism to myocardial and endothelial cells at varying extents during the acute infection with viremia, even if the course remains subclinical. The best evidence in support of this concept is demonstrated in pediatric cases with self-limited, chilblain like acral purpuric lesions (41). These children remain otherwise asymptomatic and, interestingly, often test negative for SARS-CoV-2 in nasopharyngeal samples (41). Nonetheless, the presence of intact virions in endothelial cells was shown by electron microscopy in skin biopsy samples from the lesions. Furthermore, the immunohistochemical staining of these tissues was positive for SARS-CoV-2 (42). The affected tissue also had the presence of lymphocytic T and B cell infiltrates and occasional microthrombi suggesting ongoing antiviral immune response and microangiopathic damage (42). Currently, there is no clear understanding of the viral propagation for certain tissues or whether this represents the strength of local immune surveillance.

## Myocarditis and COVID-19

### Literature on Viral and Post-viral Myocarditis

The International Society and Federation of Cardiology of the World Health Organization defines myocarditis as an inflammation of the heart myocardium, diagnosed by established histological immunological and immunohistochemical criteria (43). Myocarditis affects ~22 per 100,000 people a year. Most cases are due to viral etiologies including single-stranded RNA viruses—coronavirus, parvovirus, enterovirus, and coxsackievirus (44). The diagnosis and correlations to viral load can be challenging as the clinical course varies, ranging from asymptomatic to fulminant (45). Regardless of the type of virus or severity of acute infection, there is a bimodal pattern of the natural course. Based on the results of preclinical models, during the initial phase, viral propagation within the myocardial cells leads to cell damage and exposure of cryptic antigens such as cardiac myosin into the systemic circulation that can lead to breakage of tolerance possibly from molecular mimicry (46). In normal immunocompetent hosts, there is an expectation of viral clearance and resolved myocarditis; at that point, the cascading events are due, in part, to the totality of viral clearance and/or the cellular damage left by the viral infection. It is important to note, even after full clinical recovery from the acute infection, viral genomic material can be present within the cardiac cells without detectable viral propagation (47). Therefore, clinical recovery may not be a true indication of normalized immune homeostasis. In fact, the presence of even relatively small numbers of infected or damaged cells can be sufficient to sustain immune activation and tissue inflammation (48). Evidence suggests that the persistence of these perturbations can eventually lead to dilated cardiomyopathy and fibrosis (49). Regardless of the patient's status (i.e., with or without clinical or subclinical infection), some, but not all patients, can cascade into a second phase when there is heightened inflammation within the cardiac tissue even after complete clearance of viral infection, i.e., post-infectious myocarditis. This phase usually follows the prominent involvement of adaptive immunity and development of autoantibodies in genetically-susceptible individuals (50) and has elements of immune dysregulation including the primary role of over-activated monocytes/macrophages (51, 52). The early steps in the pathogenesis of immune reactivation leading to cytokine storm are not well-understood. It has been shown that some autoantibodies can cause damage and lead to the death of cardiomyocytes (53). These patients may present with rapidly-progressive shock, heart failure, arrhythmia, and abnormal heart MRI with increased T2 signal (18, 45). The diagnostic work-up in adult patients usually includes coronary angiography to rule out ischemic injury (54). While often clinically impractical in children, the diagnostic gold standard is based on endomyocardial biopsy providing histopathology evidence of lymphocytic infiltrates as well as any PCR evidence for viral infection (45, 55).

### Cardiovascular Involvement of COVID-19

Clinically, the cardiovascular complications of COVID-19 in adults have been diverse. At one end of the spectrum, there are patients with evidence of subclinical myocarditis. In a recent report, 26 previously-healthy athletes (mean age 19.5 years, and 57.7% males) with PCR-confirmed SARS-CoV-2 were studied by cardiac magnetic resonance imaging (CMR) at 11 to 52 days post-diagnosis (56). Two had mild shortness of breath and the rest of the cohort were asymptomatic. The EKG, transthoracic echocardiogram, and serum troponin I levels were all normal. Out of 26, 12 had evidence of myocarditis by late gadolinium enhancement (LGE) that was with (4/12) or without (8/12) increased T2 signal. In general, it has been recognized that COVID-19 can cause acute thromboembolic events or arrhythmias that may result in cardiac arrest and sudden death even among asymptomatic patients without preexistent cardiovascular conditions (57, 58). Most often COVID-19 associated cardiac compromise is recognized among patients requiring hospital care. In a study involving 191 inpatients, about 1:5 had increased cardiac enzymes and evidence of heart failure (59). Overall, the level of myocardial compromise appears to have three major determinants: (1) the presence of pre-existing cardiovascular disease, for whom over 50% develop elevated cardiac enzymes and often progress to malignant arrhythmia and cardiac failure (59); (2) intensity of viral load manifesting with high troponin levels and severe viral myocarditis (60); and (3) inappropriate inflammatory response resulting in acute respiratory distress syndrome, multi-organ failure and disseminated intravascular coagulation that share some features with macrophage activation syndrome (MAS) (61).

### Suggested View on MIS-C as a Post-viral Myocarditis Cause of CAAs

Based on available literature on viral myocarditis including those by coronaviruses (62), SARS-CoV-2 has the potential to cause post-infectious myocarditis, and the underlying pathogenesis is expected to be similar across all ages. The increased risk of CAAs in children is likely a reflection of the microanatomy during growth (63) impacting the mechanical strength of the internal elastic lamina. It may explain why giant aneurysms are seen mostly in infants. Interestingly, there have been 27 reported cases in adults with a MIS-C like presentation, referred to as MIS-A (64). It remains to be seen if confounding risk factors, such as Ehlers-Danlos syndrome or atherosclerosis, will contribute to the development of vascular aneurysms among adults (65).

## Analysis of MIS-C as a Post-Infectious Myocarditis and Systemic Vasculitis Affecting the Coronary Arteries—Comparisons to Other Known Pediatric Rheumatologic Conditions

### Demographics of MIS-C

The overall proportion of children with SARS-CoV-2 infection presenting with MIS-C is largely unknown. However, according to data as of October 15, 2020, reported by the Centers for Disease Control and Prevention (CDC), there were 1,097 confirmed cases and 20 deaths related to MIS-C, with 98% testing positive for antibodies to SARS-CoV-2 (66). The mean age was 8 years, and the male-to-female ratio was 54:46. The majority (75%) of cases were in Hispanic or African-American individuals, correlating with the overall racial distribution of COVID-19 cases in the US (66). These findings were very similar to those reported globally, as summarized in Table 1 (5, 10).

### Similarities in the Clinical Presentation of MIS-C and Kawasaki Disease

The clinical presentation of MIS-C shares many similarities with Kawasaki disease (KD), and most notably, for causing CAA in almost 1 in 5 patients (Table 1) (67, 68). Both conditions typically present with a persistent fever > 38.0°C for a median of 5 days followed by other non-specific symptoms such as vomiting, abdominal pain, and diarrhea (22). Similar to presenting symptoms in KD, a large number of children with MIS-C also presented with a rash, conjunctival injection, oropharyngeal erythema, and lip swelling/redness (22, 23, 69). A majority of MIS-C patients presenting thus far met the criteria for either typical or atypical KD (22, 23, 70). The diagnostic criteria for complete KD include: (1) Fever for > 5 days plus > 4 other features: (2) (a) generalized maculopapular rash; (b) bulbar conjunctival injection; (c) erythema of the lips, throat, and tongue; (d) desquamation of hands/feet, and (e) cervical lymphadenopathy > 1.5 cm (67, 71).

### MIS-C Presents in a Spectrum of Clinical Severity That Can Mimic Those Caused by Bacterial Constituents

The clinical course of MIS-C, however, is widely variable as compared to KD. In contrast to KD, patients with MIS-C present more frequently with a septic shock-like syndrome (68), and ~2 of 3 cases often require vasopressors for marked hypotension (Table 1). According to early data compiled by the CDC, 48% of patients were managed in the intensive care unit (ICU) with vasopressors (72). Similar to KD (68), MIS-C patients can develop transient left ventricular dilation, systolic dysfunction, pericardial effusion, and/or mitral regurgitation. In fact, 3 of 4 patients with MIS-C show findings suggestive of myocarditis, including elevated troponin and brain natriuretic peptide levels, and echocardiogram findings of decreased myocardial function as evidenced by a low ejection fraction (Table 1). One comparative study indicates that MIS-C more frequently spares the coronary arteries than KD, but is more often associated with a myocardial injury that is evidenced by global dysfunction or more subtle changes in diastolic function and ventricular strain seen on echocardiography (73). Many of those presenting with signs of shock and myocardial injury require ICU-level care (16, 74, 75). Some of these findings can also be seen in Kawasaki Disease Shock Syndrome (KDSS), which can present with shock-like clinical features including poor perfusion, markedly elevated inflammatory markers, mitral regurgitation, prolonged myocardial dysfunction, and coronary artery aneurysms distinguishing it from typical KD, although at a significantly lower incidence than MIS-C (76).

In addition to presenting similar to KDSS, MIS-C also shares some clinical similarities with toxic shock syndrome (TSS), which often necessitates prophylactic treatment with empiric antibiotics. TSS is a toxin-mediated illness caused by both *Staphylococcus aureus* and *Streptococcus pyogenes*, presenting most commonly with fever, rash, hypotension, a desquamating rash, and multisystem organ involvement (77). Patients present with TSS at a mean age of 11.4 years old compared to the mean age of 8 in MIS-C (66, 78). The mortality for TSS is also much higher than mortality for MIS-C, with around 5–10% in streptococcal TSS and 3–5% in staphylococcal TSS (77).

### Lessons Learned From Acute Rheumatic Fever: The Classic Model of Post-bacterial Carditis

Acute rheumatic fever/carditis (ARF/ARC) is a classic example of post-infectious heart disease and is caused by exposure to group A β hemolytic streptococcus (GABHS). The diagnosis of ARC is based on Jones criteria (79) composed of minor criteria (reflecting the activation of the innate immunity resulting in fever, joint pain, increased CRP and in some cases prolongation of PR interval on EKG) and major criteria that signifies the actual immune-mediated pathology in the target organs (carditis, arthritis, skin, subcutaneous nodule and chorea). It is often considered as a generalized vasculitis involving both small and large vessels in multiple organ systems, but it is not associated with CAAs (80). Furthermore, ARC does not involve myocardial or intramyocardial connective tissue and clinically does not result in elevated cardiac enzymes such as troponin (81, 82), or depressed ventricular contractility or systolic function (83), which are clinical markers of myocarditis. The cardiac dysfunction is secondary to hemodynamic changes from valvular damage resulting from endocardial inflammation of the connective tissue over the mitral and aortic valves (83). Studies of the autoantibodies involved in causing ARC target endocardium as well as cardiac myosin and laminin on the cell surface and laminar basement membrane of the endocardium/endothelium leading to upregulation of VCAM-1 and infiltration of CD4+ T cells into the valve (84, 85). Of note, unlike ARC, MIS-C is not associated with rheumatic valvulitis.

## Activation of IL-1β in MIS-C and MIS-C-Like Post-Infectious Conditions of Children

### Extrapolating Immunopathogenesis of MIS-C Based on the Assumption That KD Is Also Within the Spectrum of Post-viral Myocarditis

MIS-C provides overwhelmingly strong evidence for a viral trigger (e.g., SARS-CoV-2) leading to coronary artery aneurysms. Although KD is suspected to be triggered by a viral infection (86), research remains inconclusive, given the lack of reproducibility of a single pathogen resulting in KD (87, 88). It is possible that a group of agents rather than a single agent may be the true representation for the etiology of KD. Based on this view, we believe MIS-C and KD represent a spectrum of post-viral myocarditis with shared principles for immunopathogenesis.

### The Central Role of the Innate Immune System and IL-1 Pathway

Within that framework, the conditions detailed in Table 2, including MIS-C and KD, share two major elements: the presence of endothelial damage (85, 89–92) and upregulation of the IL-1β pathway (7, 23, 93, 94). IL-1 is a pleiotropic cytokine that is produced along with IL-18 upon activation of the NF-kB pathway through the engagement of toll-like receptors (TLR) expressed on endothelial cells and other cells involved in the innate immune response (99). It is as a pro-cytokine that requires enzymatic cleavage by caspase 1 upon activation of intracellular inflammasome complexes that include nod-like receptor family pyrin domain-containing protein 3 (NLRP3) (99). Furthermore, activation of antigen-presenting cells (APCs)—i.e., macrophages and dendritic cells—by paracrine mechanisms and proinflammatory signaling cascade, results in primed adaptive immunity and downstream activation of effector T cells (100). In the presence of a recent viral insult, and perhaps delayed clearance of viral genome or proteins, the IL12/IFN-γ axis is reinforced along with the feedback loop to homing killer lymphocytes to the heart (101). In addition to activation of CD8^+^ cytotoxic T lymphocytes (CTL), IFN-γ also upregulates HLA expression of the host tissue cells that may result in increased sensitivity of immune damage. In addition, the presence of dendritic cells, and monocytes/macrophages as well as CTL whose activities are likely to be further damaging the vessel wall (68, 102).

**Table 2:** Stratifying common properties among MIS-C and MIS-C like conditions (7, 23, 85, 89–98).

	**MIS-C**	**KD**	**KDTSS**	**TSS**	**ARC**
IL-1β	**↑**	**↑**	**↑**	**↑**	**↑**
CBC changes for WBC & platelets	**↑**	**↑**	**↑**	**↑**	**↑**
Tissue findings					
Myocarditis	**+**	**+**	**+**	**–**	**–**
Vasculopathy	**+**	**+**	**+**	**+**	**+**
Role of					
Superantigen	**+/−**	**+/−**	**+/−**	**+**	**+**
Oligoclonal antibody	**?**	**+**	**+**	**–**	**+**
Complement activation	**+/−**	**+/−**	**+/−**	**+**	**+**

*↑ means elevated, + means present, − means absent, ? means unknown, +/− means sometimes present*.

Clinically, these conditions share a similar phenotype, often characterized by a precipitous onset of fever for 7 to 10 days and a self-limited course. Laboratory findings usually demonstrate increased proinflammatory cytokines and more specifically upregulated IL-1β along with increased levels of downstream IL-6, IL-8, TNF-α (103). Uniformly, these patients have hematologic changes with leukocytosis and thrombocytosis along with increased levels of serum acute phase reactants as discussed in detail below (104). The outcomes of the activated IL-1β pathway has been well-studied through the autoinflammatory syndromes derived from monogenic mutations of inflammasomes. This includes cryopyrin-associated periodic fever syndrome (CAPS) due to gain of function mutation of the NLRP3 (105). In general, when secreted, IL-1β remains mostly in the tissue microenvironment and serum levels of free IL-1β remain low as reported on patients with MIS-C (7, 20, 23). Therefore, assays based on RNA expression are preferred. Children with MIS-C invariably show increased IL-18, which is produced along with IL-1β and converted from pro-IL-18 to IL-18 through activation of caspase 1 (106). IL-18 augments levels and functional activities of IFN-γ (106). Similar to KD (107), MIS-C has evidence of cytokine storm and increased IL-18 and IFN-γ (23), but it does not fulfill the criteria of MAS. Given the importance of IL-1 in the pathogenesis of the development of coronary artery aneurysms in KD (93), treatment of MIS-C with an IL-1 receptor antagonist (Anakinra) became one of the preferred therapeutical agents to control inflammation and reduce the risk of tissue damage as discussed below.

### Can the Activation of NLRP3 Be a Trigger for MIS-C?

We believe the role of NLRP3 and upregulation of IL-1β may play a key role in the pathogenesis for MIS-C and MIS-C like post-infectious conditions. Viral proteins including SARS-CoV 3a protein can directly activate the NLRP3 inflammasome in lipopolysaccharide-primed macrophages (108). Furthermore, recent genome-wide studies have shown SNP association of the inositol 1,4,5-triphosphate 3 kinase (ITPKC) gene with increased risk of KD and CAAs due to loss of function of ITPKC, which is a negative regulator of NLRP3 (109). Similar genetic risk factors are likely to play a role in MIS-C. Several mechanisms can result in NLRP3 activation and immune dysregulation seen in KD and MIS-C. For instance, microbe-associated molecular patterns (MAMPs) are well-known to upregulate IL-1β and can be found in 76.7% of serum samples from patients with KD (110). Among those, M protein from GABHS is a potent inducer of NLRP3 (111). Even subclinical presence of bacterial components from streptococcal species including GABS and *S. pneumoniae*, and *S. aureus* that carry molecular components with shared homology to superantigens (112) may be enough to disrupt immune homeostasis toward inducing a hyperinflammatory state. Superantigens are potent inducers of IL-1 and can induce antigen-independent polyclonal activation of T and B cells as well as activate complement by the alternative pathway (95, 96). The role of superantigens has been of great interest in KD (113). The supporting evidence includes findings in the preclinical models (114) using *Lactobacillus casei*, S. aureus derived TSS toxin (115), or FK565 (116). Interestingly, SARS-CoV-2 was shown to possess intrinsic superantigen-like qualities. Structure-based computational models suggest the mutation of a spike protein at D839, found in the European strain of SARS-CoV-2, enables the spike protein to bind to a T cell receptor and T cell costimulatory molecule CD28. Interestingly, this motif shares similarities to Staphylococcal endotoxin B (SEB) (95, 96). This finding is significant in its correlation to the demographics of patients presenting with MIS-C.

### Cascading Immune Reactions Following IL-1β

Upon activation of IL-1β, and through paracrine mechanisms, there is a downstream increase in expression of IL-6, IL-8, TNF- α, IL-10, IL-1RA, CXCL10 (93, 99). This results in increased expression of adhesion molecules on vascular endothelial cells. The histopathologic findings on autopsy tissues at varying time points after onset of KD demonstrate the neutrophilic infiltrate in the internal elastic lamina of the arterial wall that plays a major role in causing vascular damage secondary to degranulation of proteolytic enzymes during the first 10 days of illness (117). This is followed by an influx of CD68+ macrophages, CD3+CD4+ and CD3+CD8+ T lymphocytes, CD20+ B lymphocytes, and IgA or IgM-producing plasma cells that are essential for injury to the endothelial wall and subsequent development of CAAs (117). There have been reports of MIS-C with evidence of low C3 and C4 (17, 118). The mechanism of complement activation by either the lectin pathway or classical complement pathway remains unknown.

### Role of Specific Immunity

The role of specific immunity in KD is strongly supported by the evidence of an association with specific HLA genotypes that are likely to contribute to the differences in incidence between the Eastern and Western hemispheres (119); similar correlations are anticipated for MIS-C. Another important finding is the presence of IgA^+^ oligoclonal plasma cells in the vascular wall (117) and immune complexes in the blood (120) among patients with KD, suggesting the involvement of antigens in the development of vasculitis seen in KD (121, 122). The development of anti-cardiac myosin autoantibodies has been reported in cases that may have heart involvement in KD (97). During B cell maturation, isotype switch to IgA takes place in mucosal-associated lymphoid tissue (MALT). As patients with KD often present with gastroenteritis, the gut-associated lymphoid tissue (GALT) is likely involved (123). IgA can activate complement and augment inflammation upon coupling with antigen and mannose-binding protein (MBP) (123). MIS-C often presents with diarrhea (124), and similar mechanisms of IgA involvement may occur. Finally, about 40% of patients with viral myocarditis are found to have anti cardiac antibodies (125, 126), probably, due in part to exposure of cryptic antigens upon viral tropism. Interestingly, their symptom-free relatives can also have similar autoantibodies suggesting the involvement of genetic factors influencing central tolerance. Recently, preliminary data suggested the presence of SARS-CoV-2 antibody cross-reactivity with host α-myosin (98). This is a similar mechanism to that initially described in landmark studies on ARC regarding the role of molecular mimicry between microbial antigens (i.e., M protein and the group A carbohydrate epitope of GABHS) and cardiac myosin in genetically susceptible individuals (127). Further, cardiac myosin exposed from damaged tissues can directly bind to toll-like receptor-2 (TLR-2) with downstream activation of NFκB and production of proinflammatory cytokines including IL-1β (128).

### Knowledge Gap on the Status of Anti-myocardial Antibodies in MIS-C to Predict Long Term Outcomes

It is important to note that acute viral myocarditis and KD rarely reoccur; so far, similar observations hold for MIS-C as well. This has been attributed to the role of adaptive immune response preventing re-infection (129). Chronic post-infectious cardiac conditions such as ARC and dilated cardiomyopathy (DCM) are associated with anti-myocardial antibodies to myosin (130) and troponin. ARC progressively worsens over time with evidence of tissue injury characterized by fibrotic valvular changes, lymphocytic infiltrate, and Aschoff bodies, particularly after recurrent exposures to GABHS. Likewise, DCM may progress upon reexposure to viral agents (49, 131). Long term outcomes of cardiac complications post COVID-19 are currently unknown. Further studies are needed to help expand current understanding (126, 132) of the roles of clonal expansion, cryptic antigens, molecular mimicry, superantigens and complement activation, as deeper insights evolve on the status of interferon pathways (133), and regulatory lymphocytes in breakage of immune tolerance by SARS-CoV-2.

## Clinical Aspects and Management of MIS-C

### The Utmost Importance in Patient Care Is the Prompt Recognition and Treatment of MIS-C

**Figure 1:**
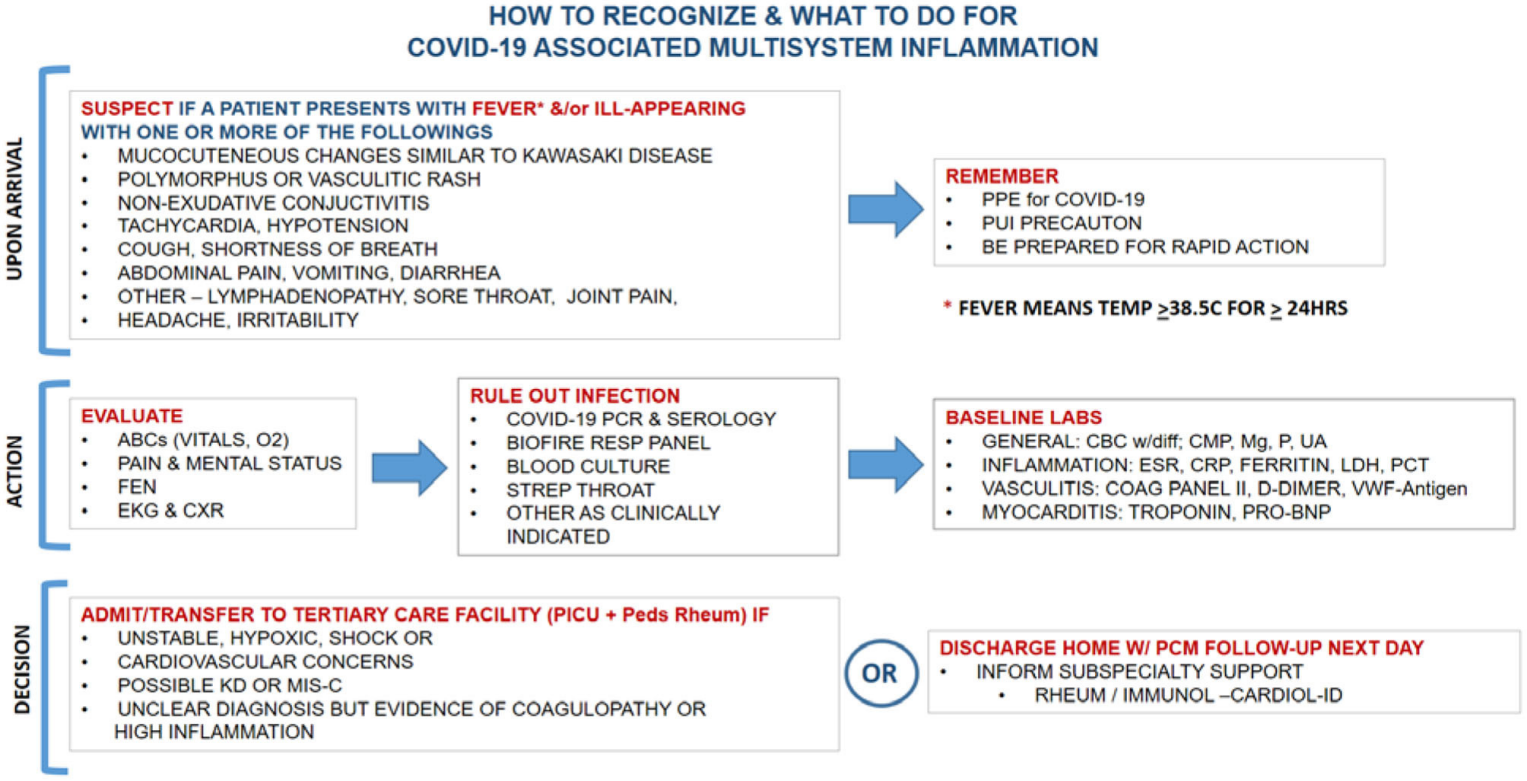
Initial recognition and management of MIS-C.

Given that viral tropism of SARS-CoV-2 to cardiac tissue is seen in patients presenting with MIS-C, the emergent management of MIS-C is critical. Figure 1 summarizes suggested steps in the management of an ill child suspected of MIS-C or like conditions presenting to the hospital.

### Laboratory Investigation of MIS-C Is Extensive, but Longitudinal Tracking Is Essential for Tailored Treatment

As shown in Table 3, laboratory surveillance on peripheral blood samples can be grouped under 4 major domains: (1) routine survey of complete blood count (CBC) and comprehensive metabolic panel (CMP), (2) inflammatory markers, (3) cardiac, and (4) coagulation and endothelial markers. Importantly, there is often an associated increase in cardiac specific markers—troponin (troponin I and troponin T)—as evidence of ongoing direct cellular injury to the myocardium and subsequent myocarditis. Unbound troponin present in the cytoplasm is released into the bloodstream upon an acute ischemic injury to cardiomyocytes within 2 to 4 h, peaking in 12 h and returning to normal levels in 7 to 14 days (134). Therefore, the presence of, and in some cases, continuing upward trend in troponin levels is concerning (74). B-type natriuretic peptide (BNP) hormone is secreted primarily from cardiac myocytes in the ventricles in response to wall tension and stretching as a prohormone (proBNP) that is cleaved into biologically active C-terminal hormone and biologically inert N-terminal proBNP (NT-proBNP) (135). In MIS-C, increases in the levels of these peptides can be associated with fluid overload manifesting as peripheral edema. Significant elevations of BNP and related peptides suggest worsening myocardial interstitial edema and subsequent vasculopathy secondary to capillary leak in the myocardium (18). In conjunction with resolving systemic inflammation in patients with MIS-C, BNP usually improves within 2 days, but troponin may remain high for a longer period until the regenerative repair of injured cells. Overall, the serum pro-BNP and troponin levels in MIS-C are usually higher than those found in KD and reflect the extent of vasculopathy and overall damage to cardiomyocytes.

**Table 3:** Reported laboratory findings in patients with MIS-C (6, 7, 11–23).

**Parameters**	**Mean (range)**	**% elevated ↑ or decreased ↓ (*n*)**
**Complete blood count**		
White blood cell count (×10^9^/L)	13.4 (3.95–42.8)	NR
Lymphocytes (×10^9^ cells/L)	0.94 (0.12–6.44)	↓ 88.4% (114/129)
Neutrophils (×10^9^ cells/L)	13.0 (3.3–36.4)	↑ 100% (104)
Hemoglobin (g/dL)	10.6 (5.3–18.1)	↓ 81.5% (44/54)
Platelets (×10^9^ cells/L)	210.1 (69–892)	↑ 30.8% (4/13), ↓ 47.4% (9/19)
**Cardiac inflammatory markers**		
BNP (pg/mL)	3624.7 (16–17814)	↑ 94.4% (67/71)
NT-proBNP (pg/L)	7129.1 (155–59291)	↑ 97.7% (84/86)
Troponin I (ng/L)	394.7 (10–6900)	↑ 93.4% (57/61)
Troponin T (ng/L)	80.0 (6–1771)	↑ 86.4% (19/22)
**Systemic inflammatory markers**		
d-Dimer (mcg/mL)	3.97 (0.35–19.33)	↑ 88.2% (165/187)
C-reactive protein (mg/mL)	222.6 (2.96–456)	↑ 100% (162/162)
ESR (mm/h)	57.3 (21–130)	↑ 100% (46/46)
Procalcitonin (ng/mL)	82.5 (0.1–127)	↑ 89.5% (111/124)
Lactate dehydrogenase (U/L)	354.7 (178–4087)	↑ 100% (71/71)
Fibrinogen (mg/dL)	628.2 (328–948)	↑ 94.4% (51/54)
Ferritin (ng/mL)	645.0 (153–2010)	↑ 96.6% (85/88)
**Chemistry**		
Sodium (mmol/L)	133.2 (116–141)	↓ 95.2% (20/21)
Aspartate transaminase (U/L)	41.1 (15–151)	↑ 66.7% (4/6)
Alanine transaminase (U/L)	43.3 (6–257)	↑ 60% (15/25)
Albumin (g/dL)	3.3 (1.6–4.7)	↓ 83.6% (56/67)
**Cytokines**
Interleukin-1 (pg/mL)	0.8 (0.4–1.6)	↑ 0% (0/4)
Interleukin-2 (pg/mL)	NR	↑ 100% (8/8)
Interleukin-6 (pg/mL)	169.8 (3.1–1366)	↑ 64.9% (50/77)
Interleukin-8 (pg/mL)	45.2 (25.1–149)	↑ 50.0% (6/12)
TNF-α (pg/mL)	54.8 (44.4–68.8)	↑ 100% (4/4)
**Coagulation**		
Prothrombin time (s)	16.2 (12.1–22.1)	↑ 90.9%
Partial thromboplastin time (s)	37.9 (27.5–85)	↑ 87.5%

MIS-C patients also present with evidence of systemic hyperinflammation demonstrated by increased levels of acute-phase reactants including C-reactive protein (CRP), fibrinogen, ferritin, procalcitonin, which are released primarily by the liver in response to proinflammatory cytokines (23, 136). While procalcitonin may initially be high, it typically downtrends within hours of its peak unless there is an ongoing bacterial superinfection. Erythrocyte sedimentation rate (ESR) reflects the composition of serum proteins—particularly levels of fibrinogen—and is often significantly elevated. In addition to the elevated troponin and BNP as discussed above, the presence of increased D-dimer and Von Willebrand factor antigen in MIS-C also suggests vasculopathy. This is evident as a clinical manifestation of peripheral edema as well the tendency of fluid overload and is an important warning for increased predisposition to thromboembolic events to the heart, lungs, and brain as seen in patients with SARS-CoV-2 (18, 74). Although increased ferritin, D-dimer, lactate dehydrogenase (LDH), soluble IL-2 receptor (sIL-2R), IL-18 (23, 136) may signify emerging macrophage activation syndrome, full-blown MAS has not been a common finding among the children with MIS-C. In addition to IL-18, there has been consensus on serum cytokine levels for IL-6 among children with MIS-C (19) suggesting activation of both the NF-kB pathway of innate immunity and downstream inflammatory signaling cascades. Children with MIS-C often develop severe lymphopenia that may be secondary to increased levels of IL-6, which is known to downregulate lymphopoiesis through effects on hematopoietic stem cells (104, 137). While serum IL1 levels are not increased in the majority of cases, this does not dismiss the central role for IL-1 as discussed above.

## Treatment Choices and Reported Patient Outcomes

The management of MIS-C patients requires admission to tertiary care centers offering pediatric intensive care unit (PICU) and subspecialty services. Initiation of treatment should be prompt to minimize risk for cardiac decompensation and development of CAA as shown in Table 1.

**Figure 2:**
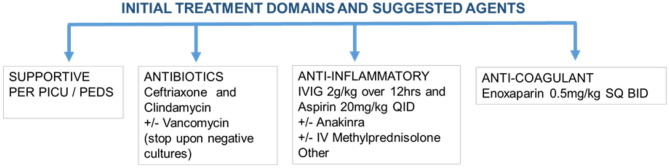
Management of patients presenting with MIS-C.

In general, the rationale for current treatment protocols can be grouped under 4 major categories as detailed in Figure 2. The first domain involves supportive care to maintain fluid-electrolyte balance, as well as blood pressure and respiratory support. The second domain involves empirically treating potential bacterial infections with broad-spectrum antibiotics particularly in those presenting in shock.

### Initial Treatment of MIS-C Involves High Dose IVIG and Aspirin

The anti-inflammatory management is aimed to reduce tissue inflammation to prevent or limit the progression of CAAs and cardiac injury, which is most optimally managed by a multi-disciplinary team. Important in the overall treatment of MIS-C is the patient's past medical history of frequent infections, family history, and preexisting comorbid conditions. Table 4 summarizes the published treatment regimens between April and August 2020. Out of 255 cases, 198 were treated using high dose intravenous immunoglobulin (IVIG) at 2 g/kg and aspirin, which relies heavily on the treatment protocol for KD in the management and prevention of CAAs. This management is now a part of the standard of care per published guidelines by the American College of Rheumatology (138). IVIG is an FDA approved blood product composed of purified serum immunoglobulin G protein. Each batch requires the processing of serum from an average of 1,000 to 15,000 donors by the pharmaceutical companies (139). It provides passive immunity to many infectious agents including common coronaviruses that may share epitope specificity with SARS-CoV-2, as well as bacterial agents and endotoxins (including SEB). Furthermore, IVIG serves as an immunomodulator of both innate and adaptive immunity by multiple mechanisms (140).

**Table 4:** Summary of agents used in published reports (6, 7, 11–23) for Treatment of MIS-C.

**Treatment**	**% Usage (*n*)**
Intravenous immunoglobulin (IVIG)	77.7% (198/255)
2nd Dose IVIG	17.1% (19/111)
Steroids	61.6% (157/255)
Aspirin	41.8% (71/170)
Anakinra	9.6% (24/251)
Tocilizumab	11.0% (28/255)
Infliximab	3.2% (8/251)
Anticoagulation (enoxaparin, etc.)	63.3 (133/210)
Antibiotics	87.7 (50/57)

Following the administration of IVIG to children with KD, the response to treatment is determined based on the rapid resolution of fever. Similarly, in MIS-C patients treated with IVIG, rapid resolution of symptoms has been commonly reported (14). This correlates with a significant reduction in cytokines, monocytes, macrophages, neutrophils, activated T cells, as well as an increase in natural killer (NK) cells (141). It is standard of care to repeat IVIG in 2–3 days in KD patients if there is treatment resistance, which correlates to an unresolved fever. A similar treatment protocol has been suggested for MIS-C. It is important to infuse IVIG slowly over 8 to 12 h to avoid substantial fluid shifts that may result in pulmonary edema and increased pro-BNP levels that may require treatment with diuretics.

Salicylate, the active ingredient of the aspirin (ASA), is derived from willow bark and is one of the oldest known medications dating back to the time of Hippocrates to 400 BCE (142). It also has anti-inflammatory, antipyretic, and antiplatelet properties by inhibiting cyclooxygenase (COX) enzyme and as a result, synthesis of lipid mediators: thromboxane, prostacyclin, and prostaglandin. These belong to a diverse family of pleiotropic and short-lived mediators generated from arachidonic acid moieties of the cell membrane and exert biological activities on many cell types including platelets and endothelial cells (143). ASA is the prototype non-steroidal anti-inflammatory drug (NSAID) that is also used in the treatment of rheumatic fever and KD (144, 145). Traditionally, patients first receive high dose ASA followed by dose adjustment to exert a primarily anti-platelet effect upon resolution of fever. Although ASA toxicity can rarely occur during the treatment of KD or rheumatic fever/carditis, it has not so far been reported in patients with MIS-C.

### A Low Threshold to Use Steroids in the Treatment of MIS-C

Corticosteroids are the cornerstone agents used in rheumatology to control systemic inflammation through a wide range of biological effects on many cell types by downregulating the transcription of pro-inflammatory cells in their signaling pathways. Downregulation of these pathways results in a decrease in the release of many pro-inflammatory cytokines (IL-1β, TNF-α, IL-6 and multiple other interleukins) (146), as well as, inhibit co-stimulatory molecules involved in the activation and differentiation of neutrophils, antigen-presenting cells, and lymphocytes (147). Corticosteroids also affect somatic and endothelial cells by downregulating adhesion molecules, and accumulation of neutrophils (148), inhibits platelet adherence (149) and activities of T and B lymphocytes (148). Initially, intravenous methylprednisolone is preferred, particularly during cytokine storm, and can be slowly transitioned and tapered to daily oral prednisone. It is important to note, initial reports had discouraged steroid use for the treatment of MIS-C and KD, as well as COVID-19, SARS-CoV, and MERS-CoV (150). During the SARS-CoV and MERS-CoV outbreaks, it was thought that steroids delayed viral clearance and increased the risk of secondary infections (150). Additionally, steroids can cause hypertension, hyperglycemia, increased risk for thrombosis, and most importantly, infection. In the recent decade, corticosteroids are commonly used in KD and have been shown to improve coronary artery abnormalities as well as a decreased duration of symptoms, and are commonly used as adjunctive therapy (151). In concurrent use with IVIG, steroids have resulted in rapid recovery in those patients presenting with severe features of MIS-C (11). The benefits of dexamethasone were recently reported for mechanically ventilated patients with COVID-19 (152) and have recently been recommended by the WHO in patients with severe or critical symptoms (153). Given these findings, steroids should be strongly considered in the treatment of MIS-C. However, it is equally important to have a tailored treatment for each patient guided by a multi-disciplinary team rather than following fixed protocols.

### Emerging Treatment Protocols of MIS-C Using Biological Response Modifiers

Biological response modifier drugs (BRMD) are a new class of therapeutical agents composed of recombinant human monoclonal antibodies or receptor antagonists. There have been many FDA approved BRMDs used to treat a wide variety of autoimmune conditions (154). Although BRMDs have good safety profiles, they are contraindicated in patients with active infections; tuberculosis, in particular, must also be ruled out before administering.

### An Anti-IL-1 Antagonist Is the Preferred First-Line Biological Response Modifier for the Treatment of MIS-C

Anakinra is an IL-1 receptor antagonist that downregulates the downstream proinflammatory cascade events secondary to IL-1. IL-1 serves as the primary immunomodulator of the proinflammatory cytokine storm (155). There have been several case reports documenting the benefit of Anakinra in pediatric vasculitis including KD (156), Henoch Schonlein Purpura (157), and connective tissue diseases (155). The beneficial effects in these studies were attributed to the downregulation of adhesion molecules on endothelial cells, inhibiting neutrophil activation, and degranulation leading to vascular wall damage. This is likely to reduce the development and progression of CAAs (93). In fact, in preclinical studies on KD, Anakinra was shown to reduce the development of coronary artery aneurysms and myocarditis (158). Anakinra is FDA approved for children with neonatal-onset multisystem inflammatory disease (NOMID) and adults with rheumatoid arthritis (159). The usual pediatric dose is 1–2 mg/kg/day and can be titrated up to 8 mg/kg/day and given as a subcutaneous injection. It comes as a prefilled syringe and the common adult dose for everyday use is 100 mg contained in one syringe (160). Anakinra was demonstrated as safe and efficacious in a small pilot study on COVID-19 patients (161). In this study, the max dose was 5 mg/kg intravenously BID and was shown to be tolerated well. These doses are higher than what is typically used in MAS (162). This encouraged usage of high doses of Anakinra on MIS-C patients given the extent of inflammation involved. Anakinra is the drug of choice given its favorable safety profile and short half-life of 4–6 h and should be considered during the initial therapy to reduce the magnitude and duration of the proinflammatory immune response. However, a multi-disciplinary team should be involved in the management of MIS-C with Anakinra to assess treatment efficacy and make dose adjustments.

### Targeting IL-6 for Treatment of MIS-C

Tocilizumab is a recombinant IL-6 receptor antagonist that downregulates the paracrine and autocrine effects of IL-6, a major proinflammatory cytokine (163) and therapeutic target for MIS-C. The serum IL-6 levels, which are consistently elevated on presentation along with down-trending CRP and ESR, and up-trending absolute lymphocyte count can be useful markers in assessing treatment response. Inhibition of IL-6 by Tocilizumab prevents downstream events including differentiation of CD4^+^ T cells and inhibition of T regulatory cells (163). As a result, Tocilizumab preserves homeostasis and may prevent disruption of immunological tolerance important in the pathogenesis of many autoimmune and autoinflammatory diseases (164). IL-1β and TNF-α play a large role in the activation of IL-6, further lending to the importance of IL-1 blockade with Anakinra (165). Tocilizumab is FDA approved for children 2 years and above for systemic juvenile idiopathic arthritis (166), which shares a similar cytokine cascade as MIS-C. Tocilizumab is available either as a subcutaneous injection (162 mg/syringe) or IV infusion. It is important to note that Tocilizumab can cause liver failure, leukopenia, and hypercholesteremia, and thus is important to screen these markers before and during treatment (163). The initial pilot studies on Tocilizumab in the treatment of serious COVID-19 infections, especially those with acute respiratory distress syndrome (ARDS) were promising (167). However, a recent phase III double-blinded clinical trial on COVID-19 patients with an ongoing active infection who were treated with Tocilizumab did not show additional clinical benefit compared to those receiving the current standard of care (168). In addition, the results of a small study of 4 patients undergoing Tocilizumab therapy suggested a possible increase in the risk of formation of coronary artery aneurysms (169). Regardless, the blockage of IL-6 is a promising target for the treatment of MIS-C and warrants further research on its efficacy and safety profile.

### Targeting TNF-α for Treatment of MIS-C

Infliximab is a TNF-α blocker that may also serve an important role in the treatment of MIS-C. Also involved in the clinical manifestations seen in Kawasaki disease, TNF-α serves an important role in the pathogenesis of MIS-C. TNF-α is thought to be a key player in proinflammatory cytokine production and platelet activation, and involved in the development of many chronic autoinflammatory diseases (170). Thus, because of its significant role in the activation of cytokine pathways, TNF-α remains a key biological target that can be utilized as a treatment option for those presenting with MIS-C. Infliximab is sometimes used in IVIG-resistant KD also some vasculitis (Behcet's disease, deficiency of adenosine deaminase 2). Inspiring from the fact that TNF-α blocker is an effective treatment modality for Crohn's disease, there has been some early success in the treatment of a patient with MIS-C and comorbid Crohn's disease (171). Currently, there is a lack of experience using combination anti-IL1 and anti-TNF-α agents. Future studies may be warranted to determine if a combined agent approach maximizes the efficacy of treatment while minimizing the adverse effects.

### Follow-Up Assessment of MIS-C for Treatment Response and Disease Damage

**Figure 3:**
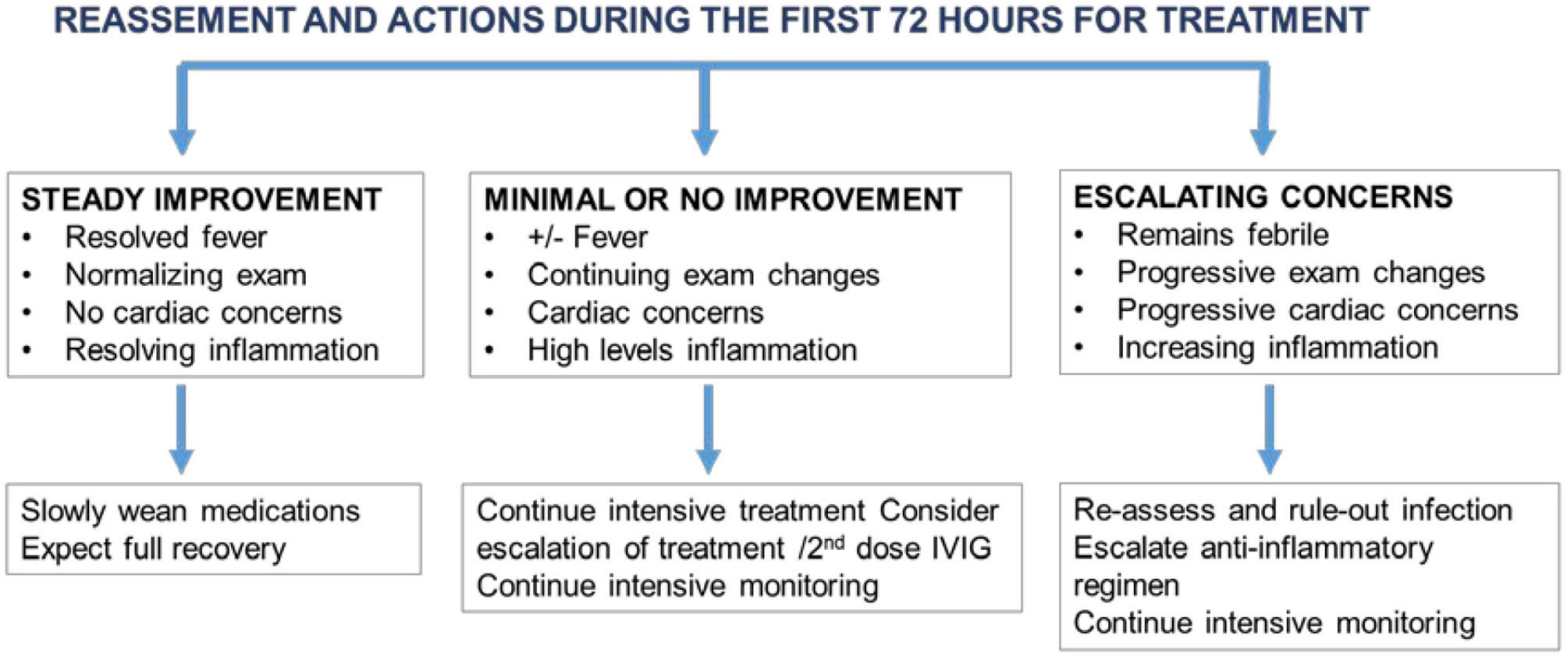
Management algorithm based on the patient's clinical status and response to initial treatment.

Patients with MIS-C require real-time patient assessment and treatment adjustments particularly in the first few days of diagnosis. Based on the initial response, treatment decisions can be modified as shown in Figure 3.

At the present time, the follow-up recommendations for patients with MIS-C are still evolving. MIS-C patients may require a slower taper of anti-inflammatory medications than is typical for KD patients. Cardiology reassessment and imaging frequency are often geared toward the type and severity of presentation. For those with a more classic myocarditis presentation that includes diminished function, patients may undergo regular cardiac testing that often includes serial electrocardiography, echocardiography, and rhythm monitoring, with consideration of cardiac MRI and exercise stress testing as clinically indicated. For those with a presentation more typical for KD (e.g., primarily CAAs as the cardiac manifestation), providers may consider adopting KD management guidelines (167). Accordingly, during the acute phase of KD, the echocardiogram is performed upon presentation, then repeated serially out to 6–8 weeks. If the child has an abnormal echocardiogram at the time of diagnosis, then the follow-up schedule and repeat echocardiogram become more frequent based on the risk level for potential myocardial infarction (172). Given all that is still unknown about MIS-C, we suggest more frequent echocardiography assessment during the first week of presentation, then spacing the frequency once the clinical trajectory is better established. Providers may also consider adding a cardiac MRI around 3 months post-MIS-C to assess for evidence of fibrosis and to help stratify long-term risk. We emphasize the importance of multidisciplinary collaboration during the convalescent phase.

## Summary

MIS-C is an emerging phenomenon that first presented in April 2020 amid a global pandemic. In this report, we analyzed MIS-C as a post-viral myocarditis in the setting of a history of infection with SARS-CoV-2. We also presented the literature review that supports shared similarities between MIS-C and other hyperinflammatory conditions including KD, TSS, and ARC for clinical and laboratory manifestations. Evidence suggests upregulation of IL-1β pathway and activation of endothelial cells are likely to be the key determinants in disease pathogenesis among these conditions. The diagnosis of MIS-C can be challenging given the variability of presentation. Providers should have a high clinical suspicion of MIS-C during the ongoing COVID-19 pandemic and include troponin in the initial laboratories. A multi-specialty approach and prompt intervention are essential to improve patient outcomes. Currently, the treatment protocol for MIS-C is similar to that used for KD and involves IVIG and aspirin. Also, for the given severity of MIS-C, corticosteroids, and biologic response modifies such as Anakinra (IL-1 receptor antagonist) should be considered under the guidance of rheumatology consultation. The goal is to control and eliminate inflammation as quickly as possible before the onset of tissue damage (i.e., CAA). This requires real-time patient assessment and tailored treatment.

The limitations to this review include paucity of knowledge on immunopathogenesis of MIS-C, as well as, risk factors for disease severity and damage. It is our view that, MIS-C is a prototype of post-viral myocarditis affecting a selected subpopulation of infected that has become more identifiable due to the sheer numbers of patients involved during the pandemic. It remains to be confirmed if MIS-C and KD share overlapping etiopathogenesis. Under the current circumstances, as a common sense, we like to suggest, post-COVID-19, patients to remain vigilant and avoid secondary exposures that may disturb borderline homeostasis toward immune-dysregulation and cytokine storm in a form of MIS-C. As many aspects of MIS-C remain undiscovered, collective and global efforts are needed for fast-tracked patient-centered research, including the status of immune-memory and autoimmune serology to predict long term outcomes.

## Author Contributions

JCM contributed to the article by extensive literature review, drafting, and editing the paper. JWM contributed in depth cardiology aspects and critical review. MWC contributed in immunopathogenesis of autoimmune heart disease, autoantibodies and critical review. OYJ contributed in defining a framework of the content and concepts involved as well as drafting and editing the paper. All authors contributed to the article and approved the submitted version.

## Conflict of Interest

The authors declare that the research was conducted in the absence of any commercial or financial relationships that could be construed as a potential conflict of interest.

## Abbreviations

ABC: Airway Breathing Circulation
ARC: Acute Rheumatic Carditis
ARF: Acute Rheumatic Fever
BRMD: Biological Response Modifier Drugs
CAA: Coronary Artery Aneurysm
CBC w/diff: Complete Blood Count with white blood cell differential
CMP: Complete Metabolic Panel
CRP: C-Reactive Protein
COAG Panel II: Coagulation panel
CXR: Chest x-ray
EKG: Electrocardiogram
ESR: Erythrocyte Sedimentation Rate
FEN: Fluids Electrolytes Nutrition
IVIG: Intravenous Immunoglobulin
KD: Kawasaki Disease
LDH: Lactate Dehydrogenase
MAS: Macrophage Activation Syndrome
Mg: Magnesium
MIS-C: Multisystem Inflammatory Syndrome in Children
P: Phosphorus
PCT: Procalcitonin
PPE: Personal Protective Equipment
PRO-BNP: N-Terminal prohormone of Brain Natriuretic Peptide
PUI: Patient Under Investigation
Rheum-Immunol-Cardio-ID: Rheumatology-Immunology-Cardiology-Infectious Disease
TNF-α: Tumor Necrosis Factor α
TSS: Toxic Shock Syndrome
UA: Urinalysis
VWF Antigen: Von Willebrand Factor Antigen.
